# Autotetraploid Induction of Three A-Genome Wild Peanut Species, *Arachis cardenasii*, *A. correntina*, and *A. diogoi*

**DOI:** 10.3390/genes15030303

**Published:** 2024-02-27

**Authors:** Robert W. Suppa, Ryan J. Andres, Jeffrey C. Dunne, Ramsey F. Arram, Thomas B. Morgan, Hsuan Chen

**Affiliations:** 1 Pairwise, Research Triangle Park, NC 27709, USA; rsuppa@pairwise.com; 2 Department of Crop and Soil Science, North Carolina State University, Raleigh, NC 27695, USA; rjandres@ncsu.edu (R.J.A.); jcdunne@ncsu.edu (J.C.D.); 3 Department of Horticultural Science, North Carolina State University, Raleigh, NC 27695, USA; rfarram@ncsu.edu (R.F.A.); tbmorgan@ncsu.edu (T.B.M.)

**Keywords:** polyploidy, ploidy manipulation, genome doubling, autotetraploid, hybrid barrier, wild germplasm, peanut breeding, secondary germplasm, cytogenetics

## Abstract

A-genome *Arachis* species (AA; 2n = 2x = 20) are commonly used as secondary germplasm sources in cultivated peanut breeding, *Arachis hypogaea* L. (AABB; 2n = 4x = 40), for the introgression of various biotic and abiotic stress resistance genes. Genome doubling is critical to overcoming the hybridization barrier of infertility that arises from ploidy-level differences between wild germplasm and cultivated peanuts. To develop improved genome doubling methods, four trials of various concentrations of the mitotic inhibitor treatments colchicine, oryzalin, and trifluralin were tested on the seedlings and seeds of three A-genome species, *A. cardenasii*, *A. correntina*, and *A. diogoi*. A total of 494 seeds/seedlings were treated in the present four trials, with trials 1 to 3 including different concentrations of the three chemical treatments on seedlings, and trial 4 focusing on the treatment period of 5 mM colchicine solution treatment of seeds. A small number of tetraploids were produced from the colchicine and oryzalin gel treatments of seedlings, but all these tetraploid seedlings reverted to diploid or mixoploid states within six months of treatment. In contrast, the 6-h colchicine solution treatment of seeds showed the highest tetraploid conversion rate (6–13% of total treated seeds or 25–40% of surviving seedlings), and the tetraploid plants were repeatedly tested as stable tetraploids. In addition, visibly and statistically larger leaves and flowers were produced by the tetraploid versions of these three species compared to their diploid versions. As a result, stable tetraploid plants of each A-genome species were produced, and a 5 mM colchicine seed treatment is recommended for A-genome and related wild *Arachis* species genome doubling.

## 1. Introduction

Peanut, *Arachis hypogaea* L., is an important row crop covering hundreds of thousands of hectares and yielding nearly three million tons annually in the US and over 50 million tons worldwide [1,2]. Peanuts can be consumed as whole nuts or processed into peanut butter, oil, or meal [1]. The peanut yield can be hindered by various biotic stresses, such as nematodes, *Sclerotinia* blight, leaf spots, or tomato spotted wilt virus (TSWV), causing hundreds of millions of dollars of annual losses worldwide [3]. Cultivated peanut varieties could be improved by introducing beneficial traits from wild *Arachis* species if interspecific hybridizations are accessible and hybrid barriers (or hybrid infertility) can be broken. Many known resistance traits have been observed in wild peanut species; however, the interspecific hybridization between cultivated and wild counterparts frequently results in hybrid incompact or sterile hybrid progeny because of the difference in genome type and ploidy [3].

*Arachis* species contain diverse genome types and ploidies. Most *Arachis* species are diploids (2n = 2x = 20), categorized into subgenomes A, B, D, F, G, or K [4]. Direct hybrids of diploid *Arachis* species from different subgenomes are typically sterile or have low fertility [4]. The genus *Arachis* originates from South America, and *A. hypogaea* (2n = 4x = 40, AABB) is an allotetraploid hypothesized to be derived from a hybrid of two species, *A. ipaënsis* (2n = 2x = 20, BB) and *A. duranensis* (2n = 2x = 20, AA) [5]. The hypothesized hybrid ancestor became a fertile allotetraploid via unreduced gametes or endoduplication [4]. Evidence of genome sequencing, chromosome structure, and geographic analysis further support this hypothesis [6,7]. Because of the AABB genome of cultivated peanuts, wild *Arachis* species with AA or BB genomes are potential germplasm sources for cultivated peanut breeding. These strategies have been developed with multiple goals in mind. The wild species contain many resistance genes that are beneficial to *A. hypogaea*. Still, they also possess morphological traits that may be unique to the species and desirable or undesirable depending on the effect. An introgression strategy must accommodate these traits and determine the optimal method of creating the ideal introgression parent.

Conquering the hybrid barrier of infertility is the first challenge in the introgression of beneficial traits. Knowledge of the genome type and ploidy differences between wild germplasm species and cultivated peanuts has led to multiple accomplishments in introgressing beneficial traits, typically involving ploidy manipulation steps [8]. One method involves directly crossing the diploid wild species and tetraploid cultivated peanut, resulting in triploid progeny and doubling the triploid genome to a hexaploid state. The hexaploid shows some level of fertility and can backcross to tetraploid cultivated peanuts or be allowed to self-fertilize for several generations, eventually reverting to a fertile tetraploid state that carries some wild-species-derived DNA [3,8]. The second pathway creates a diploid AB hybrid by crossing A-genome and B-genome parents and using genome doubling methods to develop an AABB amphidiploid compatible with *A. hypogaea* [3,8]. A third pathway uses genome doubling to create an autotetraploid from the two wild species, AAAA or BBBB, before crossing to make the compatible AABB hybrid or directly hybridizing the autotetraploid (AAAA or BBBB) to the cultivated peanut and then backcrossing for several generations until the progeny genome reverts to the AABB genome [3]. These introgression strategies have demonstrated that genome doubling methods are critical to overcoming the ploidy barriers when using wild *Arachis* germplasm.

Mitotic inhibitor chemicals are applied to plant cells or meristems to create genome-doubled cells and individual plants [9,10]. Mitotic inhibitors break down the microtubules in cells while cells are dividing, making the cell unable to separate chromosomes into daughter cells during mitosis [10]. As a result, incomplete mitosis culminates in a doubled genome [9,10]. Plant tissue usually experiences stress and shows temporary morphological abnormality or death after chemical treatment [11]. After recovery, surviving cells can restart mitosis and divide as tetraploid cells. To receive fully tetraploid plants, the mitotic inhibition process is usually applied to plant tissue materials with meristematic tissues, including seeds [11,12], axillary meristems [13,14,15], and seedling apical meristems [16]. The methods of application, chemical types, and concentrations have been known to have varying effectiveness between species [10]; therefore, optimizing an established protocol may be necessary for each species or plant material.

According to Ref. [3], mitotic inhibitors, namely colchicine, oryzalin, and trifluralin, are the most used for genome doubling in plant breeding [17]. Their effectiveness can vary by species and tissue type. For example, oryzalin showed better efficiency than colchicine [12]; however, colchicine showed better results in *Cannabis sativa* chromosome doubling [13]. Trifluralin used in genome doubling is comparatively newer than the other two, and it was preferred in some cases due to its reduced toxicity [17]. In *Arachis* species, the published mitotic inhibitor treatment methods are all based on colchicine [3]. The first is a treatment for steam cuttings, where the apex of a cutting is submerged in a liquid solution containing the dissolved chemical [3,5]. The second treatment method involves exposing a young shoot, particularly the meristem, to the mitotic inhibitor [3]. The third method is to submerge seeds that have recently germinated in a liquid solution with colchicine [3,8]. Genome doubling methods in *Arachis* species have been reported to be inefficient and difficult to repeat [3], while improving genome doubling methods would make introgression easier and more consistent. Some published methods have reported successful genome doubling, suggesting that *Arachis* species are generally susceptible to colchicine; however, their susceptibilities to other commonly used mitotic inhibitors, like oryzalin and trifluralin, remain unknown. Research on *Arachis* species polyploid manipulation efficiency regarding the chemical type, concentration, and type of plant tissue applied to will help peanut breeders to introgress desirable genes from diverse wild germplasms, especially diploid wild species.

*Arachis* species with A subgenomes have been used to introgress genes into cultivated *A. hypogaea* (AABB). *A. cardenasii*, known for its resistance to root-knot nematode, was used to develop root-knot-nematode-resistant cultivars [4,18]. Using the amphidiploid pathway, *A. cardenasii* (AA) was crossed first with *A. diogoi* (AA) to stack desirable traits, and the resulting hybrid was crossed to *A. batizocoi* (KK but closely related to BB), followed by genome doubling via colchicine treatment (AABB) before being successfully hybridized with cultivated peanut [18]. This hybrid was backcrossed with *A. hypogaea* for additional selection, resulting in the ‘COAN’ cultivar [18]. The yield of ‘COAN’ was determined to be similar to that of check cultivars. Testing against checks with no nematicide application resulted in a 225% greater yield, successfully demonstrating its nematode resistance [18]. Since ‘COAN’, numerous successful peanut cultivars have been released that incorporate this nematode resistance, including NemaTAM, Tifguard, TifNV-High O/L, and NemaTAM II [3,19,20,21]. In addition to nematode resistance, *A. cardenasii* has been shown to be an excellent source of resistance to leaf spots. Beginning in the 1960s, a series of *A. cardenasii* introgression lines were produced via the triploid–hexaploid–tetraploid route at North Carolina State University [22,23,24]. This leaf spot resistance has been incorporated into the majority of Virginia-type peanut cultivars grown in the United States [25] and has been used extensively throughout the world as a source of leaf spot resistance [26]. Taken together, these hybridizations were one of the earliest successful introgression attempts and laid the foundation for the use of these methods with other wild *Arachis* species.

Another A-genome species, *A. correntina*, was also reported to resist various diseases [4]. To introgress leaf spot disease and tomato spotted wilt resistance into cultivated peanuts, *A. correntina* was used as the A-genome parent in a hybrid with *A. ipaënsis* (BB) [4,27]. The sterile diploid hybrid (AB) had fertility restored through the colchicine genome doubling treatment to a tetraploid state (AABB), which was able to hybridize with cultivated lines [27]. Seeds harvested from these new allotetraploids were evaluated for their disease resistance potential [27]. This *A. correntina* hybrid was determined to resist late leaf spot disease and tomato spotted wilt virus, making it a potentially valuable genetic resource for introgression into cultivated lines [27].

In addition to being used in interspecific hybrids for nematode resistance, *A. diogoi* (A genome) has been used to develop introgression lines because of its resistance to leaf spot, tomato spotted wilt virus, and root-knot nematode [4]. This species also possesses a variety of other disease and pest resistances, making it a species with a useful genetic background for introgression lines [4]. In previous research, *A. diogoi* introgression lines were created via the triploid–hexaploid–tetraploid route at North Carolina State University [28]. This hybridization led to the development of a population with strong leaf spot resistance, proving that this species is a useful germplasm for peanut breeding [29].

Knowing the importance of genome doubling in introgressing beneficial traits from wild germplasm into cultivated peanuts, the objective of this research is to establish a protocol for the genome doubling of A-genome *Arachis* species and creating tetraploid plant material of selected A-genome species for future breeding processes. In this study, three A genome *Arachis* species, *A. cardenasii*, *A. correntina*, and *A. diogoi*, with demonstrated value in peanut breeding, were chosen to establish genome doubling methods. A total of four trials of chemical treatment methods were tested. The first, second, and third trials included different concentrations of the three chemical treatments, including three types of mitotic inhibitors, colchicine, oryzalin, and trifluralin, on seedling apical meristems, and the fourth trial focused on the treatment period of 5 mM colchicine solution treatment in seeds.

## 2. Materials and Methods

### 2.1. Plant Materials

Seeds of three A-genome *Arachis* species, *A. cardenasii* (PI 262141), *A. correntina* (PI 262808), and *A. diogoi* (PI 276235), were used. Seeds from all species were obtained from the North Carolina State Peanut Breeding & Genetics Wild Species Collection. Seeds were sown, and plants were grown in a greenhouse maintained at 21 °C at the Horticultural Science Field Lab, Raleigh, NC (35°47′28.9″ N 78°41′53.6″ W) from February 2022 to June 2023. Seeds were sown in 2-inch pots before or after the chemical treatment under a mist bench for a 15-s duration at 30-min intervals. After the mitotic inhibitor treatments, plants were maintained under natural light to maintain growth until they had fully recovered from the chemical treatments. After flow cytometry tests, selected plants were transferred to a 50 × 36 × 11 cm heavy duty plastic vented tray (Kadon Corp., Dayton, OH, USA) with a 1:1:1 mix of sand (R.L. Bradsher Contracting Inc., Raleigh, NC, USA), sterile topsoil blends (R.L. Bradsher Contracting Inc., Raleigh, NC, USA), and Sungro potting mix (Seba Beach, AB, Canada). Granular slow-release fertilizer (Florikan CRF with Nutricote Total, 18-6-8 (N-P-K), 270 Day 3-Stage, Sarasota, FL, USA) was applied at 2.5 g per container. In total, four trials of chemical treatment were applied; the first three trials were solid agar tube treatments (see Section 2.2), and the fourth trial was a submerged seed treatment (see Section 2.3).

### 2.2. Seedling Solid Agar Treatment

To prepare the solid agar with a mitotic inhibitor, 0.5% agar solution was prepared for the solid agar seedling treatment and then microwaved to boiling before cooling. Once the agar solution had cooled to 35 °C, the mitotic inhibitor chemicals were added separately. The filled tubes were placed in a rack in a 4 °C refrigerator to allow the gel to be fully set and stored.

The first trial contained varying concentrations of either colchicine, oryzalin, or trifluralin to make initial observations about their effectiveness in February 2022 (Table 1). The first trial treatment included 10 mM colchicine, 30 mM colchicine, 0.1 mM oryzalin, 0.3 mM oryzalin, 0.1 mM trifluralin, and 0.3 mM trifluralin. Additional trials using the solid agar treatment method were focused on optimizing a single mitotic inhibitor.

**Figure 1:**
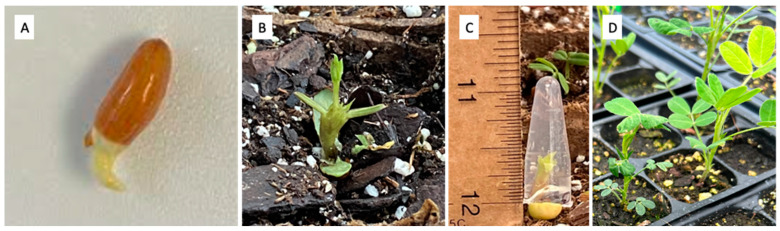
Demonstration of the solid agar mitotic inhibitor treatment in *Arachis* seedlings. (**A**) Seed germination after 72 h of 100 ppm gibberellic acid pre-treatment; (**B**) a seedling at the treatment size (1–1.5 cm) with the two developed tetrafoliate leaves removed; (**C**) a seedling under 72 h of solid agar treatment; (**D**) seven weeks after treatment, a surviving seedling showed abnormal growth with wrinkled and smaller new leaves (left) compared to untreated control (right).

In the first trial, 60 seeds of each species were sown, and every 10 seeds were directly sown in the same tray and each tray assigned to one mitotic treatment. Seedlings were considered ready for treatment when the meristem was approximately 1–1.5 cm above the cotyledons (Figure 1B). The first two tetrafoliates of each seedling were removed to ensure that the tube would fit on the meristem and that the meristem could be fully exposed to the mitotic inhibitor gel for 72 h on a shadowed bench (Figure 1C). After the mitotic inhibitor treatment, the agar gel microtubes were removed from the container, and the seedlings were rained with water.

To enhance the germination rate and the consistency of seedling growth, gibberellic acid pre-treatment was used in all trials other than the first trial. In the gibberellic acid pre-treatment, seeds of each species were placed in paper towels dampened with 100 ppm gibberellic acid in a sealed plastic container in a dark room at room temperature. Seeds were left in the container for 72 h, at which point they were removed from the container and rinsed with water. Gibberellic-acid-pre-treated seeds (Figure 1A) were planted into trays containing Sungro potting mix (Seba Beach, AB, Canada) to grow until they reached 1.5–2 cm, ready for solid agar mitotic inhibitor treatment.

The second trial was designed to improve the colchicine conversion rate through a lower concentration, 5 mM (Table 2). In May 2022, 15 seeds of each species were pre-treated with gibberellic acid and sown, resulting in 12–15 seedlings of each species for the 5 mM soil agar treatment. The third trial was designed to optimize the oryzalin treatment to improve the survival rate (Table 3). The third trial treatment included 0.1 mM oryzalin, 0.3 mM oryzalin, and 0.5 mM oryzalin. In July 2022, 70, 50, and 70 gibberellic-acid-pre-treated seeds of *A. cardenasii*, *A. correntina*, and *A. diogoi* were sown, resulting in 60, 40, and 60 seedlings readied for mitotic inhibitor treatments. The solid agar treatment method was the same as in the first trial.

Ten weeks after each mitotic inhibitor treatment trial, the number of surviving plants in each treatment was counted. Plants with new growth and standard-morphology leaves were used in the ploidy test by flow cytometry. An alive seedling that did not grow any new leaves in ten weeks was not counted as a surviving plant because such plants eventually died within a few months without further growth.

### 2.3. Pregerminated Seed Submersion Treatment

In the fourth trial, gibberellic-acid-pre-treated seeds (Figure 1A) were directly treated with mitotic inhibitor solutions. In January 2023, 70, 86, and 77 seeds of *A. cardenasii*, *A. correntina*, and *A. diogoi* were pre-treated with gibberellic acid, with the same method as in the second and third trials. Seeds were then treated with the two mitotic inhibitors, which was done by submerging the gibberellic-acid-pre-treated seeds in a liquid water solution containing either 5 mM colchicine or 0.1 mM oryzalin. Seeds were treated by placing them in a beaker containing the liquid solution treatment for the chosen treatment period (Table 4). Beakers were placed on a shaker set at 100 RPM to keep the solution mixed. The fourth trial treatments included 6 h of 5 mM colchicine, 6 h of 0.5 mM oryzalin, 12 h of 0.5 mM oryzalin, 24 h of 0.5 mM oryzalin, and 24 h of water control in March 2023 (Table 4).

At the end of the treatment period, seeds were removed and rinsed with water before immediate planting in soil trays. Seeds were grown for ten weeks to allow them to recover before the flow cytometry tests.

### 2.4. Flow Cytometry

Ploidies reflected by the DNA content of each surviving plant were tested by flow cytometry (Quantum P Ploidy Analyzer, QuantaCyte, Mullica, NJ, USA). Some surviving plants grew a few small, wrinkled, and abnormal leaves before resuming the production of normal-looking leaves, while other plants directly grew normal-looking leaves (Figure 1D). A ploidy test was applied on the first two normal-looking leaves of each seedling. For each sample, 0.5–1 cm^2^ seedling leaf tissue was chopped using a razor in 200 μL of nuclei extraction buffer (Cystain Ultraviolet Precise P Nuclei Extraction Buffer; Sysmex, Görlitz, Germany). Then, 800 μL of stain buffer (Cystain Ultraviolet Precise P Staining Buffer; Sysmex, Görlitz, Germany) was added and mixed by gently shaking the Petri dish. The chopped samples with buffers were filtered using a 50 μm gauge filter (Celltrics, Sysmex America Inc., Lincolnshire, IL, USA) and collected in a 3.5 mL plastic tube (Sarstedt Ag & Co., Nümbrecht, Germany).

Nuclei were then analyzed using the flow cytometer. Each seedling was analyzed twice using new tissue to confirm the observed ploidy. Diploid *Arachis* plant material was used for reference standard set peak placement. Seedlings were tested independently, and the placement of the peaks determined their putative ploidy. A seedling with both leaf samples testing as a tetraploid was then defined as a tetraploid. A seedling with at least one leaf sample showing a mixoploid or its two samples showing inconsistent results was defined as a mixoploid. A seedling with both leaf samples testing as a diploid was defined as a diploid. Tetraploid plants were transplanted into flat trays, and their ploidies were re-tested 4–6 months later; plants that remained tetraploid were then defined as stable tetraploids and cloned via stem cuttings.

### 2.5. Phenotypes of Diploid and Tetraploid Plants

The flower size (width) and leaflet size (length and width) were measured and compared. Each plant had ten leaves measured, when possible, with the length from apex to base and the width at the widest point of each leaflet being collected. Images of plant leaves were captured against a quarter-inch grid and were perspective-corrected before measuring. The Fiji software, a distribution of ImageJ, was used to perform perspective correction and measurements. The most significant specimens were selected for analysis. Flower size data were also collected, but a dataset of sufficient size could not be collected due to the timing of data collection in the growing season and some plants not being mature enough to produce flowers. Data analysis was performed using R, with no additional packages. A two-tailed *t*-test with was used to compare each trait of the diploid and tetraploid plants.

## 3. Results

### 3.1. Seedling Solid Agar Treatment

The first trial started with 20 seeds per treatment; however, a very low germination rate was obtained, and only 1–9 seeds germinated for each treatment tray. The low germination rate might have been because no gibberellic pre-treatment was conducted. Due to the sporadic results and low sample number, no meaningful statistics could be performed. As result, one tetraploid *A. cardenasii* was received from the 10 mM colchicine treatment and one tetraploid *A. correntina* was received from the 0.1 mM oryzalin treatment.

Generally, the seedlings of the three A-genome *Arachis* species showed high sensitivity to colchicine and almost no response to trifluralin (Table 1). Only two of the 15 colchicine-treated seedlings survived, and one was tested as a tetraploid in the first flow cytometry test. In contrast, 28 of 31 trifluralin-treated plants survived, and none of them were tested as tetraploids.

High oryzalin showed a moderate effect on the survival ratio but was not statistically significantly different from either colchicine or trifluralin. The result primarily showed that colchicine and oryzalin have better potential in genome doubling for A-genome *Arachis* seedlings, and trifluralin showed almost no effect. A reduced colchicine concentration and increased oryzalin treatment were tested in the next two trials.

The second trial was focused on a lower concentration of colchicine (5 mM) compared to the first trial and started with 12–15 seedlings of each species (Table 2). The result showed that the 5 mM colchicine treatment was highly lethal, with only two of 55 treated seedlings surviving; however, one of the two surviving seedlings was a tetraploid, and the other was a mixoploid. Both tetraploid plants reverted to mixoploids at four months after treatment. Due to the low survival rate, no meaningful statistics could be performed, but the results indicated that the 5 mM colchicine gel treatment may still be too strong for A-genome *Arachis* seedlings.

The third trial was focused on various concentrations of oryzalin gel treatment (0.1, 0.3, and 0.5 mM) on seedlings. The results were shown to be rather spontaneous. The survival rates of the three A-genome species after all three concentrations of oryzalin treatment averaged 94%, with a range of 88–100%. Unexpectedly, the survival and tetraploid conversion rates did not correlate with the oryzalin concentration. Two tested tetraploid individuals were obtained, one from the 0.1 mM oryzalin treatment and one from the 0.5 mM oryzalin treatment. Therefore, increasing the oryzalin concentration did not increase the tetraploid conversion rate. The tetraploid plant reverted to a mixoploid at four months after treatment.

### 3.2. Seed Submersion Treatment

The fourth trial focused on various concentrations of colchicine and oryzalin solutions, submerging seeds within a solution containing their respective treatment. The results are presented in Table 4. The results showed that the 6-h-colchicine treatment had the most optimal tetraploid conversion rate. In addition, the seeds showed high sensitivity to aquatic treatment, and treatments longer than 12 h resulted in high lethality. All tested seeds died in the 24-h water control and 24-h oryzalin groups. In the 12-h oryzalin treatment, 0–22% survival rates were observed, and none of the seven surviving seedlings were tetraploids. Similar survival rates were observed in the 6-h oryzalin and the 6-h colchicine treatments; however, all four seedlings tested were from the 6-h colchicine treatment. Due to the low survival rates, no statistically significant differences were detected.

However, the 6-h colchicine treatment resulted in a high tetraploid conversion rate from the surviving seedlings. For *A. cardenasii*, two of the five surviving seeds were tetraploids. For *A. correntina*, one of the four surviving seeds was a tetraploid. Likewise, for *A. diogoi*, one of the four surviving seeds was a tetraploid. The *A. diogoi* tetraploid plant was first tested as a mixoploid dominated by tetraploid nuclei in the first flow cytometry test, but was then repeatedly found to be a stable tetraploid 3, 4, and 5 months after treatment. All tetraploid plants from the colchicine treatment were repeatedly tested as tetraploids 3, 4, and 5 months after treatment.

### 3.3. Phenotypes of Diploid and Tetraploid Plants

**Figure 2:**
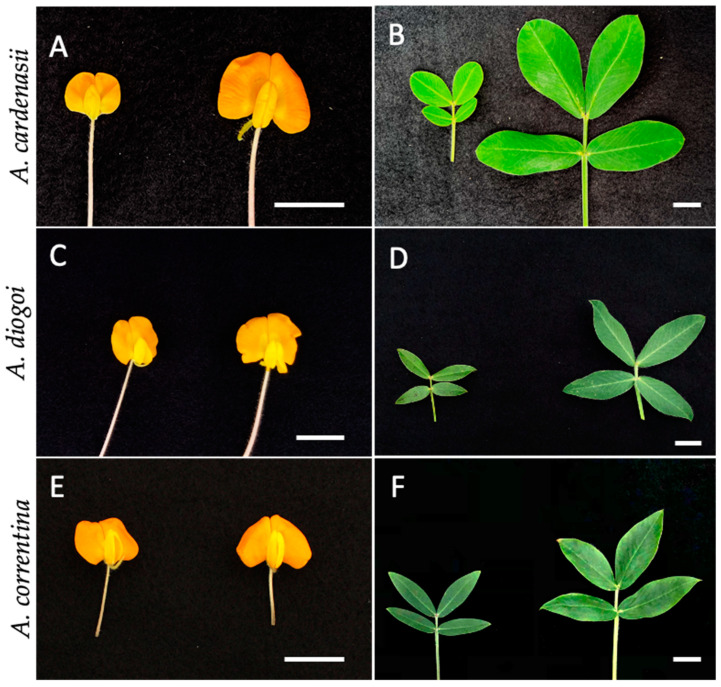
Flowers and leaves of the diploid and tetraploid versions of *A. cardenasii* (**A**,**B**), *A. diogoi* (**C**,**D**), and *A. correntina* (**E**,**F**). In each figure, the diploid plant is placed on the left, and the tetraploid plant is placed on the right. Bar = 1.5 cm.

Plants that were consistently tested as tetraploids in the four, five, and six months after treatment were defined as stable tetraploid plants. The leaf sizes and flower sizes of tetraploids and their diploid progenitors were visibly different (Figure 2). The average leaf length and width of the tetraploid versions of the three species were all significantly larger than in their diploid versions (n = 40). The flower widths of the tetraploid *A. cardenasii*, *A. correntina*, and *A. diogoi* were significantly larger than their diploid versions’ flower widths. The results are listed in Table 5 and Figure 2.

## 4. Discussion

Wild peanut has been widely used in cultivated peanut breeding for the introgression of various biotic and abiotic stress resistance genes, and genome doubling is a crucial step to overcome the hybrid barriers [3]. However, earlier publications did not discuss methods that served for *Arachis* species genome doubling. Previous wild peanut genome doubling studies provided vague information about the method and success rates with different treatments but only indicated that the conversion rate was generally low and unstable [4,30]. This study tested three commonly used mitotic inhibitors, colchicine, oryzalin, and trifluralin, on the seedlings and seeds of the three A-genome *Arachis* species. Results showed that all tested *Arachis* species were susceptible to colchicine, moderately sensitive to oryzalin, and barely responded to the trifluralin treatments. Our results indicated that colchicine and oryzalin should be the optimal mitotic inhibitors for wild peanut species. Notably, the seedlings of the three *Arachis* species were susceptible to colchicine in the first and second trials, resulting in a low survival ratio. The seedling survival rates were 0–8% in the second trial and, with the reduced colchicine concentration (5 mM) in the forth trail, the survival rate increased to 22–33%. Thus, while colchicine can be used for seedling treatment, a reduced concentration might be needed.

Mitotic inhibitor treatment in seeds performed better than in seedlings. In the first three trials, about 200 seedlings were treated, resulting in four plants that were tested as tetraploids two months after treatment. Unfortunately, all four plants reverted to mixoploids four months after treatment. Therefore, the seedling treatment had a very low success rate, and the created tetraploids tended to be unstable. This reversion led to a pivot away from the solid agar treatment method, since the research aimed to produce stable tetraploids. In contrast, all stable tetraploids were developed by the submerged seed treatments. Four tetraploid plants from these treatments were repeatedly tested as tetraploids. Lastly, the gel treatment of seedlings is substantially more labor-intensive than seed treatment. With gel treatment, seedlings need to be treated independently, whereas, in the submerged seed treatment, multiple seeds can be treated in one container. Thus, for *Arachis* species genome doubling, the seed submergence method is recommended.

However, there is room for improvement in the seed submergence method. While stable tetraploids of each tested species were produced in this study, in the fourth trial, 25–40% of surviving seeds were stable tetraploids; however, the seed survival ratio was low. We only had a 24-h water treatment as a control, and none of the control seeds survived, which was identical to the 24-h oryzalin treatment. This result indicates that the “submergence” process could be a stronger stress than the mitotic inhibitor alone. *Arachis* germinated seeds seem to be intolerant to complete liquid submergence; thus, other treatment methods might be needed. These could include increasing the oxygen level of the solution with an air pump, using mitotic-inhibitor-infused substrates to treat germinated seeds, or using a shallower solution to treat seeds without fully submerging them. With an improved survival ratio, more converted tetraploid individuals could be produced.

The morphology and phenotype can be used in a pre-screen before flow cytometry. In this study, seedlings that immediately produced a normal morphology after treatment were all tested as diploids (Figure 1D, right side), and seedlings that had an abnormal morphology after treatment (Figure 1D, left side) were more likely to be mixoploids or tetraploids. Thus, an abnormal morphology could be used as a pre-screen to reduce the labor and cost of flow cytometry. In addition, the study’s *Arachis* tetraploid plants all showed obvious morphological differences. Usually, plants with higher ploidy show significantly different morphologies, including increased leaf and flower sizes [15,17,31]. However, the morphological differences are usually not immediately visible in most plant species. An increased organ size after increased ploidy could be detected after specific repeat measurements in adult-sized plants; thus, flow cytometry is still a more trustworthy method to measure the ploidy of mitotic-inhibitor-treated plants. However, in our experiment, tetraploid or tetraploid-dominated mixoploid wild peanut plants showed immediate differences from the diploid plants. Although identifying mixoploid and tetraploid plants is almost impossible, using morphology as a pre-screen to eliminate the diploid plants could potentially reduce the laboratory work.

More ploidy manipulations of *Arachis* species are needed for breeding purposes. Other *Arachis* species with A or B genomes have also been examined to identify their usefulness in developing introgression lines. *A. duranensis* (AA), *A. stenosperma* (AA), *A. kempff-mercadoi* (AA), and *A. magna* (BB) have been reported to have a tolerance to many pests, such as silvering thrips (*Enneothrips flavens*), leafhoppers (*Empoasca fabae*), and peanut root-knot nematodes (*Meloidogyne arenaria*), offering great potential for peanut breeding [4,30,32]. The K-genome species *A. batizocoi* was also proven to be hybridizable to cultivated peanut and could be used in introgressing resistance genes from the K genome to the B genome [8,33,34]. It will be beneficial to double such species with great breeding potential for breeders to use. We recommend using the low-concentration colchicine solution treatment on the seeds of these potential species to produce germplasm for peanut cultivar development.

## 5. Conclusions

Wild peanuts have been an important germplasm source in cultivated peanut breeding for the introgression of beneficial traits. Genome doubling is critical in breaking the hybrid barrier between wild peanut species and cultivated peanuts. This research tested three mitotic inhibitors and two treatment methods in the genome doubling of three A-genome *Arachis* species. The result showed that 5 mM colchicine solution treatment of seeds could be an applicable method for A-genome wild peanut species genome doubling, with a high tetraploid conversion rate (6–13% of total treated seeds, or 25–40% of surviving seeds). The research also reports that visible morphologic differences between the diploid and tetraploid versions of the three species can be readily observed.

## Figures and Tables

**Table 1:** The first trial: mitotic inhibitor gel treatment on seedlings of three A-genome *Arachis* spp.

Species	Gel Treatment (mM)	# of Sown ^+^	# of Treated **	# of Survived *	Diploid	Mixoploid	Tetraploid
*A. cardenasii*	Colchicine 10	10	2	1	0	0	1 ***
	Colchicine 30	10	3	0	-	-	-
	Oryzalin 0.1	10	4	4	2	2	0
	Oryzalin 0.3	10	1	1	1	0	0
	Trifluralin 0.1	10	5	5	5	0	0
	Trifluralin 0.3	10	1	0	0	0	0
*A. correntina*	Colchicine 10	10	3	1	0	1	0
	Colchicine 30	10	4	0	-	-	-
	Oryzalin 0.1	10	4	4	3	0	1 ***
	Oryzalin 0.3	10	5	2	2	0	0
	Trifluralin 0.1	10	6	6	6	0	0
	Trifluralin 0.3	10	7	7	6	1	0
*A. diogoi*	Colchicine 10	10	2	0	-	-	-
	Colchicine 30	10	1	0	-	-	-
	Oryzalin 0.1	10	2	2	2	0	0
	Oryzalin 0.3	10	3	2	1	1	0
	Trifluralin 0.1	10	6	5	5	0	0
	Trifluralin 0.3	10	6	5	3	2	0

^+^ Seeds were not treated with gibberellic acid and were assigned to different treatment groups before germination. * Survival 70 days after the mitotic inhibitor gel treatment. ** Low germinated seed number resulted in low treated seed number. *** Plant tested mixoploid four months after the treatment. # = number.

**Table 2:** The second trial: 5 mM colchicine gel treatment on seedlings of three A-genome *Arachis* spp.

Spp.	# of Sown ^+^	# Treated	# Survived	Diploid	Mixoploid	Tetraploid
*A. cardenasii*	15	12	1 (8.3%)	0	1	0
*A. correntina*	15	14	0 (0%)	-	-	-
*A. diogoi*	15	15	1 (6.7%)	0	0	1 *

* Plant was subsequently tested as mixoploid four months after the treatment. ^+^ Seeds were pre-treated with gibberellic acid before mitotic inhibitor treatment. # = number.

**Table 3:** The third trial: oryzalin gel treatment on seedlings of three A-genome *Arachis* spp.

Species	Oryzalin (mM)	# Treated ^+^	# Survived	Diploid	Mixoploid	Tetraploid
*A. cardenasii*	0.1	19	18 (95%)	13	4	1 *
	0.3	20	19 (95%)	17	2	0
	0.5	20	18 (90%)	14	4	0
*A. correntina*	0.1	12	11 (92%)	10	1	0
	0.3	12	10 (83%)	9	1	0
	0.5	12	12 (100%)	11	1	0
*A. diogoi*	0.1	20	20 (100%)	15	5	0
	0.3	20	18 (90%)	17	1	0
	0.5	20	19 (95%)	17	1	1 *

* Plant was tested as mixoploid four to six months after the treatment. ^+^ Seeds were pre-treated with gibberellic acid before mitotic inhibitor treatment. # = number.

**Table 4:** The fourth trial: mitotic inhibitor solution submersion treatment of seeds.

Species	Mitotic Inhibitor *	Treated Seeds ^+^	Survived ^#^	Diploids	Mixoploids	Tetraploids
*A. cardenasii*	Colchicine 6 h	15	5 (33%) ^a^	2	1	2
	Oryzalin 6 h	15	5 (33%) ^a^	4	1	0
	Oryzalin 12 h	15	3 (20%) ^a^	0	3	0
	Oryzalin 24 h	15	0 (0%) ^a^	-	-	-
	Water 24 h	10	0 (0%) ^a^	-	-	-
*A. correntina*	Colchicine 6 h	18	4 (22%) ^a^	3	0	1
	Oryzalin 6 h	18	3 (17%) ^a^	3	0	0
	Oryzalin 12 h	18	4 (22%) ^a^	3	1	0
	Oryzalin 24 h	18	0 (0%) ^a^	-	-	-
	Water 24 h	14	0 (0%) ^a^	-	-	-
*A. diogoi*	Colchicine 6 h	16	4 (25%) ^a^	3	0	1 **
	Oryzalin 6 h	16	3 (19%) ^a^	1	0	0
	Oryzalin 12 h	16	0 (0%) ^a^	-	-	-
	Oryzalin 24 h	16	0 (0%) ^a^	-	-	-
	Water 24 h	13	0 (0%) ^a^	-	-	-

^+^ Seeds were pre-treated with gibberellic acid, and only pregerminated seeds were treated with mitotic inhibitors. * Mitotic inhibitor concentrations were 5 mM colchicine and 0.5 mM oryzalin. ** The plant was tested as a tetraploid-dominated mixoploid plant on day 70 but tested as a stable tetraploid plant, 3, 4, and 5 months after the treatment. ^#^ Due to the low survival rates, no statistically significant differences were detected.

**Table 5:** Leaf and flower size (cm) of diploid and tetraploid A-genome *Arachis* spp.

Species	Ploidy	L * Length	*p*-Value	L * Width	*p*-Value	F * Width	*p*-Value
*A. cardenasii*	2×	2.38	2.5 × 10^−10^	1.59	1.9 × 10^−14^	1.10	2.49 × 10^−3^
	4×	2.93		2.29		1.66	
*A. correntina*	2×	1.82	1.6 × 10^−27^	0.83	8.5 × 10^−25^	1.46	9.03 × 10^−3^
	4×	3.67		1.48		2.38	
*A. diogoi*	2×	2.33	2.5 × 10^−9^	1.10	5.4 × 10^−15^	1.48	9.01 × 10^−3^
	4×	3.83		1.79		1.76	

* L = leaflet, n = 40; F = flower, n = 5; Compared by two-tailed *t*-test. *p*-value from the *t*-test of each trait of the diploid and tetraploid plants.

## Data Availability

The original contributions presented in the study are included in the article, further inquiries can be directed to the corresponding author.
